# Synthesis and Radiolabelling of DOTA-Linked Glutamine Analogues with ^67,68^Ga as Markers for Increased Glutamine Metabolism in Tumour Cells

**DOI:** 10.3390/molecules18067160

**Published:** 2013-06-19

**Authors:** Paul A. Pellegrini, Nicholas R. Howell, Rachael K. Shepherd, Nigel A. Lengkeek, Elisabeth Oehlke, Andrew G. Katsifis, Ivan Greguric

**Affiliations:** Australian Nuclear Science and Technology Organisation, Locked Bag 2001 Kirrawee DC, NSW 2234, Australia

**Keywords:** PET, ^67,68^gallium, glutamine, amino acid transporter, radiochemistry, DOTA

## Abstract

DOTA-linked glutamine analogues with a C_6_- alkyl and polyethyleneglycol (PEG) chain between the chelating group and the l-glutamine moiety were synthesised and labelled with ^67,68^Ga using established methods. High yields were achieved for the radiolabelling of the molecules with both radionuclides (>90%), although conversion of the commercially available ^67^Ga-citrate to the chloride species was a requirement for consistent high radiochemical yields. The generator produced ^68^Ga was in the [^68^Ga(OH)4]^−^ form. The ^67^Ga complexes and the ^67^Ga complexes were demonstrated to be stable in PBS buffer for a week. Uptake studies were performed with longer lived ^67^Ga analogues against four tumour cell lines, as well as uptake inhibition studies against l-glutamine, and two known amino acid transporter inhibitors. Marginal uptake was exhibited in the PEG variant radio-complex, and inhibition studies indicate this uptake is via a non-targeted amino acid pathway.

## 1. Introduction

Positron Emission Tomography (PET) has rapidly become an indispensable tool for clinicians in the locating and staging of tumours, as well as measuring their response to interventional therapies. PET’s main advantages over the more established Single Photon Emission Computed Tomography (SPECT) technique, is its ability to better locate an emission due to the PET camera’s concomitant detection of the coincident 511 keV photons arising from a positron-electron annihilation, giving rise to more detailed spatial imaging. Taking advantage of this technology, the glucose mimicking tracer [^18^F]fluoro-2-deoxy-d-glucose, or [^18^F]FDG, has become incredibly popular for locating sites of cancerous activity on account of their greater glucose metabolism (Warburg Effect [1,2]). Unfortunately, [^18^F]FDG uptake is not limited to tumour cells. In areas such as the brain, and certain sites of infection and healing, [^18^F]FDG has been shown to accumulate on account of the higher rates of glucose metabolism required by the inflammatory process [3]. In fact, it has been speculated that up to 30% of actively growing tumours are [^18^F]FDG-negative [4], which highlights the need for PET tracers with greater sensitivity to tumours in these problematic areas. 

Increased amino acid utilisation is a well-established feature of tumour development and researchers have attempted to make PET tracers from them, mainly using cyclotron produced ^18^F and ^11^C radionuclides [5,6,7,8,9,10]. However, on account of the short half-lives of these radionuclides, the radiotracers must be synthesised rapidly after the proton irradiation, and if the radiochemistry is performed at a venue without a cyclotron, then the decay of the sample becomes an issue. As an alternative to this, generator based radionuclide dispensing systems are convenient and can be ‘milked’ at the convenience of the intended studies. The ^68^Ge/^68^Ga Generator is a PET radionuclide generator that is gaining popularity among the PET community, and like the SPECT limited ^99^Mo/^99m^Tc generators that have been used for decades now, it can be used in a hospital and milked at intervals for routine imaging studies and research [11]. The ^68^Ge/^68^Ga Generator produces ^68^Ga (89% abundance *β^+^*, half-life 68 min) which is a daughter-product of the ^68^Ge (half-life 270 d) adsorbed onto a column. The selectively eluted ^68^Ga can then be incorporated into radiotracers with appropriate attachment moieties, such as the 1,4,7,10-tetraazacyclododecane-1,4,7,10-tetraacetic acid (DOTA) or 1,4,7-triazacyclononane-1,4,7-triacetic acid (NOTA) macrocycles [12,13].

DOTATATE, DOTATOC and DOTANOC are three examples of targeted tracers that have become useful tools employing such macrocyclic groups that can coordinate ^68^Ga. These molecules are somatostatin receptor (SSTR) ligand analogues, and take advantage of the expression of the receptors in neuroendocrine tumours, among others [14]. Originally they were developed for SPECT agents such as ^111^In as in Octreoscan® and ^177^Lu but the translation to the PET relevant ^68^Ga has opened up application in quantification of *in vivo* receptor density as well as much improved imaging quality [15]. Angiogenesis imaging is another example of receptor targeting probes employing the ^68^Ga-DOTA and NOTA systems. The Asp-Gly-Asp (RGD) motif is well known to be recognised by the α_v_β_3_ integrin receptor which is upwardly expressed in the angiogenic process [13]. The attachment of the DOTA and NOTA systems have succesfully been employed for PET imaging, although it should be noted that the NOTA chelator has the advantage of being able to complex ^68^Ga at room temperature and thus not compromise heat sensitive macromolecules [16]. Aside from larger peptide and protein targets, ^68^Ga has been incorporated into small molecule tracers as well. These molecules have included the bifunctional chelator (BFC) approach as well as an integrated approach where the coordination sphere is inherent to the tracer. ^68^Ga integrated type imaging agents have been used for myocardial uptake [17] and bone metastases [18]. ^68^Ga- labelled small molecule tracers utilising the BFC approach have been coupled to targets such as amino acids like alanine and its derivatives [19,20], and tyrosine [21,22], prompting interest in other amino acid targets.

The amino acid glutamine has been known for over 50 years as an important requirement for the metabolic processes involved in the growth and development of proliferating tumour cells [23]. Tumour cell proliferation requires rapid synthesis of macromolecules including nucleotides, proteins and lipids. As well as being an essential component of protein structure and function, glutamine is the non-toxic ammonium vehicle between mammalian cells, effectively making it the main source of nitrogen for tumour cells. Where glucose sources may be insufficient to sustain a rate of growth, some tumour cells are able to catabolise glutamine as a source of carbon through the glutaminolytic pathway [24]. Therefore, by exploiting their increased use of glutamine transporter pathways and uptake, a radioactive glutamine analogue or mimic could act as a marker for tumour activity that could broaden the application of PET based cancer markers. Furthermore, if this agent was coupled to a generator based PET radionuclide like ^68^Ga, it could pave the way for convenient, sensitive radiopharmaceuticals independent of cyclotron production runs and proximity. Of course, considering the molecular recognition characteristics of small molecules and the conjugation of metal coordinating systems such as DOTA and NOTA, the incorporation of linkers between the biologically relevant moiety and the BFC is a requirement for the molecule to retain as much of its physiological character as possible. Generally it is a more significant issue in radio-metal tracers as the chelation groups are larger than the equivalent directly labelled halide analogues such as ^18^F and pose a greater risk of interfering with the molecular nature or recognition characteristics in biological systems.

For this study the previously reported DOTAMA-C_6_-Gln ligand **7** [25], which was developed for the magnetic resonance tumour detection using Gd^3+^ based probes, was used as it was suitable for Ga chemistry/radiochemistry. A novel polyethylene glycol (PEG) analogue, DOTAMA-PEG_2_-Gln **3**, was also synthesised in order to explore the different spatial and polar characteristics that the two different linkers imparted to the subsequent radio-gallium complexes. Of the ^67/68^Ga labelled complexes created, the ^67^Ga (half-life 3.24 d) analogues were tested *in vitro* against four tumour cell lines as well as uptake inhibition studies against l-glutamine, and the known amino acid transporter inhibitors, 2-aminobicyclo-(2,2,1)-heptane-2-carboxylic acid (BCH)–the Large Neutral Amino Acid Transport Inhibitor [26], and 2-(methylamino)isobutyric acid (MeAIB)–the system A Glutamine Transporter Inhibitor [27].

## 2. Results and Discussion

### 2.1. Synthetic Chemistry

**Scheme 1:**
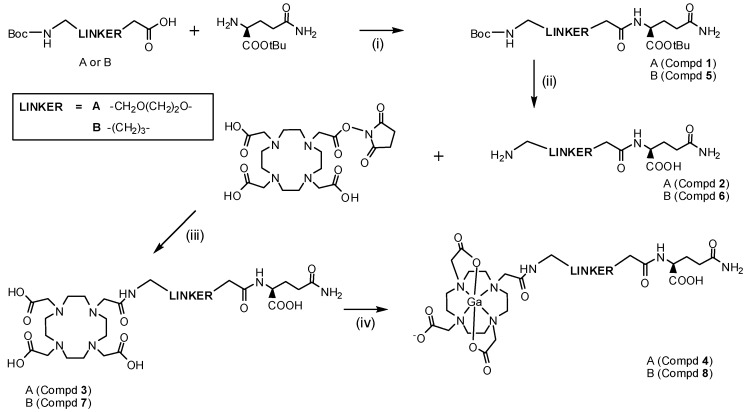
Synthetic route to the target compounds and their ^67/68^Ga analogues.

The synthetic component of this work was carried out by employing peptide coupling strategies using commercially available materials. The previously published ligand DOTAMA-C_6_-Gln **7**, was constructed in a different manner to what is outlined on account of the availability of the macrocyclic precursors. The reaction routes are outlined in Scheme 1.

*Reagents and Conditions:* (i-A) PyBOP, Et3N, DMF, DCM, RT overnight; (i-B) EDCi, HOBt, DIPEA, DMF, 0 °C, 2h, RT overnight; (ii-A&B) CF_3_COOH, RT, 2hr (iii-A&B) DIPEA, ACN, DMSO, RT overnight; (iv-A&B) ^nat^Ga(NO_3_)_3_·8 H_2_O, NH_4_OAc, H_2_O, 3 h, 80 °C or ^67^GaCl_3_, HEPES, HCl, H_2_O, 10 min 95 °C or [^68^Ga(OH)4]^−^, HEPES, HCl, H_2_O, 10 min 95 °C.

While Geninatti and co-workers started by attaching the *tert*-butyl-protected cyclam to a benzyl- protected alkyl linker to build the molecule [25], the approach used here started with the coupling of l-glutamine *t*-butyl ester hydrochloride with 6-(Boc-amino)hexanoic acid *N*-succinimidyl ester and chromatographing the crude product on silica gel. This material was hydrolysed in neat trifluoroacetic acid to remove the Boc and *t*-butyl ester groups leading to compound **6**, allowing for subsequent coupling to the DOTA NHS ester to afford the target ligand **7** with HPLC purification on an HPLC-MS with Evaporative Light Scattering (ELS) detection. The novel ligand DOTAMA-PEG_2_-Gln **3** was created in a similar fashion but employed *t-*butyloxycarbonyl-8-amino-3,6-dioxaoctanoic acid as the linker analogue. HPLC-MS purification featured in this ligand preparation as well in order to provide metal-free pure conjugates suitable for the extremely sensitive radio-metal labelling reactions. For this reason, all glassware and magnetic stir-bars were previously washed in HCl (4M). The ^nat^Ga^3+^ complexes [^nat^Ga-**4**] or [^nat^Ga-**8**] of both ligands were obtained by heating the ligands **3** and **7** with ^nat^Ga(NO_3_)_3_·8 H_2_O in buffered NH_4_OAc (pH 4.4) at 80 °C for 3 h, with the ligand in a molar excess to parallel the HPLC separation method with what would be required for the radiolabelling, as opposed to having the metal in excess. The anionic complexes were purified by HPLC-MS and were easily identifiable on account of the naturally occurring isotopic abundance ratio of ^69^Ga (60.1%) and ^71^Ga (39.9%). HR-MS further supported the identity of the complexes as well. For the integrity of the cell studies performed with the complexes [^67^Ga-**4**] and [^67^Ga-**8**], the enantiomeric purity was determined for the ligands **3** and **7** by hydrolysing 1 mg of each in 4M HCl (0.3 mL) for 2 h at 60 °C, to afford the amino acid component, glutamic acid. An aliquot of the product was chromatographed on a Phenomenex Chirex^®^ 3126 (d)-penicillamine HPLC column (150 mm × 4.6 mm) and compared with the retention times of the d*-* and l*-* enantiomers of the free amino acid. The results obtained for both ligands were >98% indicating no significant racemisation during the syntheses.

### 2.2. Radio-Chemistry

The radiochemistry of DOTA systems with various radio-metals including ^67/68^Ga is well established and testament to the versatility of these macrocyclic bifunctional chelating systems. Due to their ability to coordinate a variety of metals, they are particularly sensitive to metallic impurities and contaminants in the vessels/equipment that the chemistry is being performed in. For this reason, all of the radiochemistry, and indeed the preparative synthetic chemistry, were carried out in acid washed glassware with higher quality solvents and reagents. There is also considerable attention being given to studying the optimal conditions for such reactions to yield radiotracers with improved purities and specific radioactivities, which is particularly important for tracers that operate by receptor recognition processes. The pH of the radiolabelling reactions are extremely important for Ga. The pH must be sufficiently low so as to avoid the formation of Ga oxide and hydroxide species, but also high enough so as to deprotonate the donor pendant acid groups. The 2-[4-(2-hydroxyethyl)piperazin-1-yl]ethanesulfonic acid (HEPES) buffer system has been demonstrated as ideal for the radiolabelling reactions of ^67/68^Ga, due to its ability to provide the optimal pH range and weakly complex the Ga and thus avoid the colloidal Ga formation [28].

### 2.3. ^67^Ga Radiochemistry and Formulation

**Figure 1:**
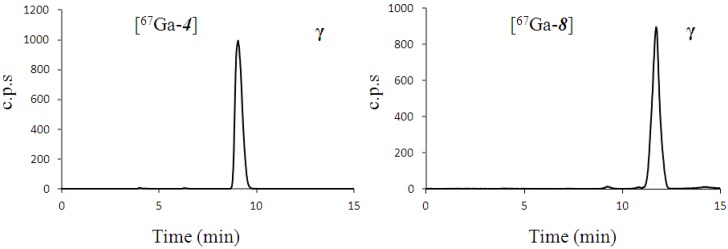
Analytical radio-trace chromatograms of complexes [^67^Ga-**4**] and [^67^Ga-**8**] produced in high yield from >200 MBq of ^67^GaCl_3_.

Initial attempts to use the ^67^Ga-citrate complex present in the commercial injection kits for the complexation reaction were unreliable, despite prior literature precedence [29,30]. Labelled complexes of both targets were produced but radiochemical yields varied from no reaction to 100% (*n* = 19). It was therefore decided to obtain ^67^GaCl_3_ from the commercial kits, employing a SiO_2_ Waters Sep-Pak^®^ based Solid Phase Extraction (SPE) procedure [31]. This source of ^67^Ga^3+^ performed as expected. Reactions were performed in small volumes, for 10 min at 95 °C in capped microcentrifuge tubes. Initial radioactivities of below 5 MBq and 1 nmol of ligand were used for method development, in the HEPES buffer system (pH 4 - 4.5). For the production of the radio-complexes for the *in vitro* studies, radioactivities of between 209 and 275 MBq were used with 15 or 25 nmol of the precursor ligands **3** and **7**. Examples of the analytical HPLC chromatograms are shown in Figure 1. Labelling yields ranged from 94.1–100% (*n* = 8) as shown in Table 1. Preparative HPLC followed, and the reaction volume was split into 2 injections to avoid overloading the semi-preparative HPLC column. 

The HPLC purified ^67^Ga-complexes showed little affinity for standard reverse-phase SPE columns or procedures. Fortunately, the anionic character of the complexes allowed them to be adsorbed onto Waters Oasis^®^ MAX Plus (Mixed-mode Anion eXchange) columns and eluted with concentrated (10×) Dulbecco’s Phopshate-Buffered Saline (DPBS) allowing simplified formulation, eliminating the use of EtOH. Overall recoveries of activity varied from 73–95% (*n* = 7) with the most significant losses occurring during the adsorption (sample loading step). Recovery of the adsorbed complex always exceeded 85%. Radio-HPLC QC of the reformulated complexes confirmed no change in the material and no formation of other products or ‘free’ ^67^Ga.

It should be noted that dilution of the HPLC fraction was a requirement for good adsorption of the radio-complexes, which lowered the methanol concentration during the loading step. Also initial attempts to elute the radio-complexes with 1 × DPBS did not perform well. The 10× concentrate provided the adequate ionic impetus for the elution, of course requiring the appropriate dilution with H_2_O to provide the products in the correct concentration DPBS buffer for the cell studies.

### 2.4. ^67^Ga complex Stability

The stability of the [^67^Ga-**4**] and [^67^Ga-**8**] -complexes in DPBS buffer at room temperature was monitored over a period of 1 week by repeated injections on a radio-HPLC system. For both complexes, no ‘free’ ^67^Ga or other decomposition products were detected, suggesting the complexes are extremely stable in buffer.

### 2.5. ^68^Ga Radiochemistry

**Figure 2:**
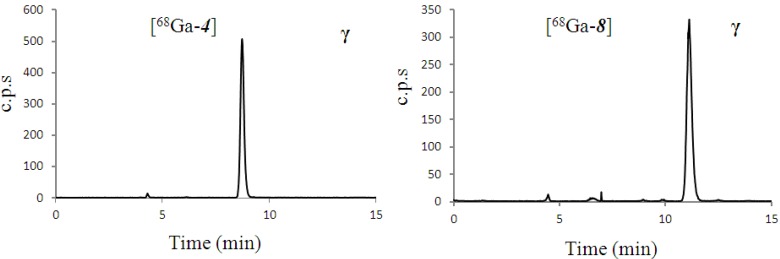
Analytical radio-trace chromatograms of complexes [^68^Ga-**4**] and [^68^Ga-**8**] produced in high yield from ~28 MBq of [^68^Ga(OH_4_)]^−^.

As a proof of concept for PET tracer development, the complexes [^68^Ga-**4**] and [^68^Ga-**8**] were synthesised using similar conditions to the ^67^Ga analogues, although starting with [^68^Ga(OH)4]^−^ from the ^68^Ge/^68^Ga Generator eluate (aqueous 0.5M KOH solution). Radiochemical yields were measured after 10 min heating at 95 °C, and were all above 90% (*n* = 6) as shown in Table 2. Reactions were initially carried out with small amounts of activity (<1 MBq) with 5 and 30 nmol of precursor ligands **3** and **7**. Reactions involving higher amounts of [^68^Ga(OH4)]^−^ were performed with less than 30 MBq, using 10 nmol of ligand. Chromatograms are shown in Figure 2. Reaction details are outlined in Table 2.

### 2.6. Cell Studies

**Figure 3:**
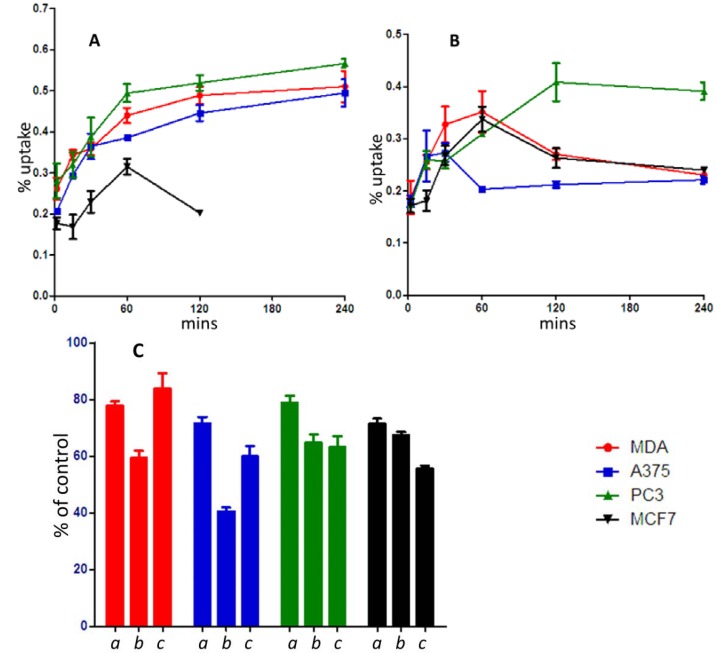
Results for cell lines MDA(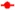), A375(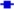), PC3(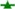) and MCF7(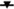). Uptake profiles for compound ^67^Ga-[**4**] (**A**) and compound ^67^Ga-[**8**] (**B**). The data is expressed as a percentage uptake of the total initial activity. Competitive inhibition of uptake of compound ^67^Ga-[**4**] (**C**) by l-glutamine (***a***), BCH (***b***), MeAIB (***c***) expressed as a percentage of the control.

Cellular uptake studies were conducted on MDA, MCF-7, A375 and PC-3 tumour cell lines with both [^67^Ga-**4**] and [^67^Ga-**8**] (Figure 3). Uptake of [^67^Ga-**4**] proceeded exponentially in MDA and PC-3 cells up until 30 min and had plateaued off by 60 min although continued to gradually increase until the assay endpoint at 240 min. In A375, [^67^Ga-**4**] uptake plateaued off at 15 min and gradually increased up until the assay endpoint. MCF-7 cells showed the lowest percentage uptake which reached a maximum at 60 min before washing out back to the baseline by 120 min. The maximum percentage of uptake [^67^Ga-**4**] was observed in the PC-3 cells. The uptake of [^67^Ga-**8**] proceeded in PC3 cells to a maximum at 120 min after which the uptake declined slightly until the assay endpoint at 240 min. In both MCF-7 and MDA cells the uptake of [^67^Ga-**8**] reached its maximum at 60 min before washing out back to the baseline by 240 min. The lowest uptake of [^67^Ga-**8**] was observed in A-375 where it plateaued off by 15 min and had washed out back to baseline by 60 min. These results show that both compounds have distinct characteristics and these characteristics differ between the cell lines used. Overall [^67^Ga-**4**] had higher rates of accumulation and retention than [^67^Ga-**8**] in all cell lines used except for MCF-7. MCF-7 cells were less tolerable of the manual manipulations required during the uptake experiment which potentially accounts for their poor uptake values. MCF-7 cells were the only cell line to show the same uptake profile for both [^67^Ga-**4**] and [^67^Ga-**8**] which was characterised by reaching the maximum percentage uptake by 60 min followed by washing out back to baseline by 120 min.

To give an indication of the uptake pathways of [^67^Ga-**4**], a competitive inhibition experiments was performed as per Bourdier *et al.* [32]. The competitors used were BCH, an inhibitor of system L amino acid transport, MeAIB, an inhibitor of system A amino acid transport and l-glutamine. The inhibition of uptake was variable across cell lines with maximum inhibition being achieved by BCH in A-375 cells. This attests to the involvement of system L transporters in the uptake of [^67^Ga-**4**] but the inability to competitively inhibit uptake with l-glutamine indicates that a major component of the uptake of this molecule is of a mode other than the targeted amino acid pathway. MCF-7 and PC-3 cells achieved equal or greater inhibition of uptake with MeAIB but the inability to inhibit uptake of [^67^Ga-**4**] with L-glutamine was consistent across all cell lines. Competitive inhibition of [^67^Ga-**8**] could not be calculated due to the propensity for the compound to wash out of the cell lines used soon after reaching its peak percentage uptake.

Having based the [^67^Ga-**8**] complex on the Gd^3+^ equivalent Gd-DOTAMA-C_6_-Gln [25], it is difficult to make comparisons between the two complexes cell uptake results. Firstly because the study used different cell lines *i.e.* HTC (rat hepatoma tissue culture, C6 rat glioma, TSA murine breast adenocarcinoma and Neuro2a (murine neuroblastoma.), and secondly as the Gd^3+^ complexes are present in concentrations much higher than for the radiotracers synthesised here. Despite these differences the ^67/68^Ga analogues seemed to be a good starting point, considering, that it was demonstrated that the alkyl linker improved cellular uptake in the HTC cells (over twice the amount of the other molecules in the study which included the non functionalised Gd-DOTAMA-C_6_-OH as well as the DOTA monoamide -glutamine variant without the alkyl linker Gd-DOTAMA-Gln [33]. As further evidence of an amino acid transporting system, it was also shown that the uptake of Gd-DOTAMA-C_6_-Gln in HTC cells was reduced significantly when in competition with increasing amounts of l-glutamine, whereas this phenomenon didn’t occur with the Gd-DOTAMA-Gln complex.

Certainly within the realms of this study, the decision to modify the linker from the C_6_- alkyl to the PEG2 appears to have improved the molecules’ uptake behaviour, despite still being very low (<1%). The PEG2 linker analogue [^67^Ga-**4**] has a greater distance between the macrocycle and glutamine moieties, which it was reasoned to reduce any steric hindrance with the glutamine’s physiological recognition processes (as well as vary the polarity). Perhaps it was still too proximal.

Of course having a bulky macrocycle appended to a very small amino acid has changed the molecule drastically, and for certain targets this may never be acceptable. It would be of great interest to obtain the HTC cells, and measure the uptake of [^67^Ga-**4**] and [^67^Ga-**8**], in an effort to make a better comparison with the Gd-DOTAMA-C_6_-Gln complex. Further to this work Stefania and co-workers varied the attachment of the glutamine group to the macrocycle linker, and found that the cationic Gd-L1 complex (attached via the α-carboxylic group doubled the cellular uptake in HTC cells in comparison to the Gd-DOTAMA-C_6_-Gln complex [34]. Kou *et al.* have synthesised the ^67/68^Ga complexes of the DOTAMA-Gln complex and performed bio distributions on Non-immunogenic fibrosarcoma cell (Nfsa) tumored C3H/HeN mice aged 8-12 weeks [30]. The uptake was rapid although quoted as ‘scarce and shielded by comparable muscle uptake.’ From that study it was concluded that ^67^Ga-DOTAMA-Gln is not a mimicker of glutamine on account of the vastly different bio-distribution profile compared to biogenic [^13^N]-l-glutamine and [^13^N]-l-glutamic acid which is understandable due to the close proximity of the glutamine and the bulky macrocyclic moiety.

In light of the results obtained and the limited reported work on these systems, it appears that radio-metal amino acid based tracers would require significant development before becoming realistic probes for targeting tumours. Appropriate chelation is required for physiological stability and these generally result in rather large groups that can impart unwanted properties to the target reducing or nullifying their biological activity. The *overall complex* must satisfy the criteria for probing a certain process or target. Of course this is not as big an issue for larger peptide/protein tracers where the metal/chelator’s effect on the macromolecule is negligible.

## 3. Experimental

### 3.1. General

All commercially available chemicals were purchased from Sigma-Aldrich, Lancaster, Macrocyclics™ and Peptides International and used without further purification. NMR spectroscopy was performed on a Bruker Avance DPX 400 operating at 400 MHz for ^1^H-NMR spectra and 100 MHz for ^13^C-NMR spectra, with shifts calibrated from the solvent signals, except for D_2_O to which ethanol was added. Low resolution mass spectrometry (LRMS) was performed on a Waters MassLynx ZQ quadrupole mass spectrometer in electrospray positive and (when suitable) negative mode using Waters MassLynx v4.1 software. High resolution mass spectrometry (HRMS) was performed on a Waters Xevo Qtof Quadrupole Time of Flight Mass Spectrometer. Preparative HPLC was performed on a Waters MassLynx HPLC-MS with Auto-purification, equipped with a Waters 600E gradient pump, 996 Photo Diode Array (PDA) detector, and a 2424 Evaporative Light Scattering Detector (ELSD) using Waters MassLynx v4.1 software. HPLC-QC purity analysis was performed on a Waters Empower2 controller system comprising of a Waters 600 pump, a Rheodyne^®^ manual injection port and Waters 2424 ELSD analyser. ELSD intensity was the basis for purity using either of the following HPLC gradient methods; Method A; Waters Atlantis^®^ T3 C-18 5 μm analytical column (4.6 × 150 mm) 1 mL/min, 0–0.5 min 100% H_2_O (0.1%TFA), 0.5–10 min, 100% H_2_O (0.1%TFA)–90% ACN/10% H_2_O (0.1% TFA); Method B; Phenomenex Synergi™ 4µ Max-RP 80A C-12 analytical column (4.6 × 150 mm) 1 mL/min, 0–0.5 min 10% ACN/ 90% H_2_O (10 mM NH_4_HCO_3_ pH 8), 0.5–10 min, 10% ACN/90% H_2_O (10 mM NH_4_HCO_3_ pH 8)–90% ACN/10% H_2_O (10 mM NH_4_HCO_3_ pH 8). ^68^Ga was produced via the ANSTO developed GAG-1 ^68^Ge/^68^Ga Generator system [35]. This material was obtained as [68Ga(OH)4]^−^ in 0.5 M aqueous KOH. Gallium-67 chloride (^67^GaCl_3_) was prepared from commercially available (Lantheus Medical Imaging^®^) ^67^Ga-citrate injection kits using a previously described method [31]. This material was obtained as ^67^GaCl_3_ in 0.1M HCl. All radiochemistry reactions involving ^67^Ga and ^68^Ga were carried out in suitably lead shielded fume hoods in plastic-ware that had been soaked in 4M HCl overnight followed by washing with de-ionised H_2_O (18.2 MΩ) and air drying. Radioactivity measurements were performed on a CAPINTEC CRC*^®^*-15R Dose Calibrator with calibration factor set to 100 for ^67^Ga and 416 for ^68^Ga. Radiochemistry HPLC-QC System: Analysis of reaction mixtures and purified products was performed on a Waters Empower2 controller system comprising of a Waters 600 pump, a Rheodyne^®^ manual injection port, a Phenomenex Synergi™ 4µ Hydro-RP 80A C-18 column (4.6 × 250 mm), an Ortec^®^ Radiation Detector (Ortec^®^ Photomultiplier Tube with Preamplifier, Model 276, operated at 1 kV through an Ortec^®^ ACEmate^TM^ pre-amplifier and bias supply system) and a Linear 200 UV detector tuned to 215 nm, both whose output data was collected with the aid of a Waters SAT-In box. The system was operated with gradient conditions; 1 mL/min, 0–1 min 5%ACN/95% H_2_O (0.1% TFA), 1–9 min 5% ACN/95% H_2_O (0.1% TFA)–90% ACN /10% H_2_O (0.1% TFA), 9–10 min 90% ACN/10% H_2_O (0.1% TFA)–5%ACN/95% H_2_O (0.1% TFA); Semi-preparative radio-HPLC system (Separation mode): Separation of radiochemical reactions was performed on Waters Empower 2 controlled system comprising of Waters 600E Controller and gradient pump, Rheodyne*^®^* manual injection port, Waters Atlantis^®^ T3 C-18 5 μm semi-preparative column (10 × 250 mm), Waters 2998 PDA (detecting at 215 and 254 nm) and a Carroll & Ramsay Associates 105S γ-detector with a sodium iodide crystal. Semi-preparative radio-HPLC system (QC mode): As per the separation mode, but an additional Waters 2424 evaporative light scattering detector was added at the end of the system. Sample injected onto this system were spiked with cold standard of either [^nat^Ga-**4**] or [^nat^Ga-**8**] for complex conformation. Sep-Pak*^®^* cartridges were activated by passage of EtOH (5 mL) followed by H_2_O (20 mL) through the column ~1 h prior to use. All reported radiochemical purities are based on peak area on the radio-trace of the HPLC chromatograms. All gamma counting was performed on a Perkin Elmer 2480 WIZARD^2^ Automatic Gamma Counter.

### 3.2. Synthesis

*Synthesis of (S)-tert-butyl 18-amino-2,2-dimethyl-4,13,18-trioxo-3,8,11-trioxa-5,14-diazaoctadecane-15-carboxylate* (**1**). *t-*Butyloxycarbonyl-8-amino-3,6-dioxaoctanoic acid·dicyclohexylamine (0.246 g, 0.553 mmol), l-glutamine *t*-butyl ester hydrochloride (0.395g, 1.65 mmol) were stirred in anhydrous DMF (30 mL), DCM (25 mL) and Et_3_N (0.4 mL). PyBOP (0.426g, 0.82 mmol) was added and the solution was stirred overnight at RT. The solution was evaporated *in vacuo*, and then EtOAc (50 mL) was added. The organic portion was washed with saturated NaHCO_3_ solution (50 mL), aqueous 5% w/v KHSO_4_ (3 × 20 mL), H_2_O (2 × 20 mL) and brine (2 × 20 mL). The resulting organic portion was dried over MgSO_4_, filtered and evaporated *in vacuo*. The crude material was dissolved in the minimum volume of 60% EtOAc/40% Pet. Ether and applied purified on silica gel and eluted with 100% EtOAc. The fraction was evaporated to dryness, yielding a clear glassy gum (0.13 g, 53%) ^1^H-NMR (d_6_-DMSO): δ (ppm)1.37–1.43 (2 × overlapped s, 18H), 1.75–1.85 (m, 1H), 1.9–2.0 (m,1H), 2.11 (t, *J* = 7.5 Hz, 2H), 3.05 (q, *J* = 6 Hz, 12Hz, 2H), 3.40 (t, *J* = 6.3 Hz, 2H), 3.5–3.62 (overlapped m, 4H), 3.91 (s, 2H), 4.16 (m, 1H), 6.7–6.8 (m, 2H), 7.30 (br s, 1H), 7.89 (d, *J* = 8 Hz, 1H); ^13^C{^1^H}NMR (d_6_-DMSO): δ (ppm) 26.58, 27.64, 28.24, 31.14, 39.6, 51.92, 69.28, 69.68, 70.24, 77.71, 80.82, 155.68, 169.51, 170.85, 173.42; ESI-MS m/z [M+H]^+^, 448.18; HR-MS (TOF MS ES^+^): 448.2659 calculated for [C_20_H_38_N_3_O_8_]^+^, found 448.2671; HPLC-ELSD purity 98.9% Method B (10.6 min).

*Synthesis of (S)-5-amino-2-(2-(2-(2-aminoethoxy)ethoxy)acetamido)-5-oxopentanoic·acid* *2CF_3_COOH* (**2**). Compound **1** (208 mg, 0.465 mmol) was stirred in neat TFA (4 mL) for 2 h at RT. The TFA was evaporated off *in vacuo*, and H_2_O (3.5 mL) was added. The aqueous portion was washed with EtOAc (4 × 5 mL), evaporated *in vacuo*, azeotroped with absolute EtOH (2 × 20 mL) then pumped dry yielding an oil (267 mg). ^1^H-NMR (D_2_O): δ (ppm) 2.0–2.1 (m, 1H), 2.2–2.3 (m, 1H), 2.41 (t, *J* = 7.3 Hz, 2H), 3.23 (t, *J* = 5 Hz, 2H), 3.75–3.81 (overlapped m, 6H), 4.16 (d, *J* = 1.4 Hz 2H), 4.46 (dd, *J* = 4.9 Hz, 9 Hz, 1H); ^13^C{^1^H}NMR (D_2_O): δ (ppm) 27.23, 31.89, 39.71, 52.81, 67.04, 69.89, 70.13, 71.05, 173.08, 175.83, 178.72; ESI-MS m/z [M+H]^+^ 292.08 [M-H]^−^ 290.19; HR-MS (TOF MS ES^+^): 292.1509 calculated for [C_11_H_22_N_3_O_6_]^+^, found 292.1512. HR-MS (TOF MS ES^−^): 290.1352 calculated for [C_11_H_20_N_3_O_6_]^+^, found 290.1344; HPLC-ELSD purity 98.2% Method A (7.8 min).

*Synthesis of (S)-2,2',2''-(10-(16-amino-13-carboxy-2,11,16-trioxo-6,9-dioxa-3,12-diazahexadecyl)-1,4,7,10-tetraazacyclododecane-1,4,7-triyl)triacetic acid DOTAMA-PEG_2_-Gln·HCOOH* (**3**). To a solution of DOTA-NHS ester (121.6 mg, 123.3 µmol) in DMSO (1.1 mL) and ACN (0.4 mL) was added compound **2** (70 mg, 187.7 µmol) in DMSO (1 mL) and DIPEA (0.15 mL) and stirred at RT overnight. The solvent was evaporated off *in vacuo* and the resulting residue was diluted in 30% ACN in H_2_O (1.5 mL) and was purified by repeated injections on a Waters ZQ HPLC-MS with a Waters Atlantis^®^ T3 C-18, 10 µm preparative column (19 × 250 mm) using the following conditions; isocratic 20 mL/min, H_2_O with 0.2% HCOOH modifier, collecting the peak at t = 8.3 min by the ES +ve chromatographic and ELSD traces. The combined fractions were reduced by rotary evaporation, and freeze dried yielding a clear solid (102.5 mg). ^1^H-NMR (D_2_O): δ (ppm) 2.0–2.1 (m, 1H), 2.1–2.16 (m, 1H), 2.40 (t, *J* = 7.4 Hz, 2H), 3.0–3.3, 3.3–3.5 (br signals 18H), 3.6–3.83 (br overlapped m, 10H), 3.83–4.12 (br s, 4H), 4.14 (br s, 2H), 4.48 (dd, *J* = 5 Hz, 9 Hz, 1H); ^13^C{^1^H}NMR (d_6_-DMSO): δ (ppm) 26.65, 28.99, 31.26, 38.58, 48.17, 48.44, 50.59, 51.23, 52.66, 53.92, 54.76, 68.72, 69.39, 69.66, 70.15, 115.29, 118.25, 157.73, 158.05, 169.41, 171.66, 171.68, 173.10, 173.56; ESI-MS m/z [M+H]^+^, [M+Na]^+^ 678.31, 700.28, [M-H]^−^ 676.33; HR-MS (TOF MS AP^+^): 678.3310 calculated for [C_27_H_48_N_7_O_13_]^+^, found 678.3344, 676.3154 calculated for [C_27_H_46_N_7_O_13_]^−^, found 676.3187; HPLC-ELSD purity 99.9% Method A (8.1 min); Chiral purity >98%.

*Synthesis of ^nat^Ga(III) DOTAMA-PEG_2_-Gln·* *CF_3_COOH* (^nat^Ga-***4***). To a stirred, buffered solution of ligand **3** (25.6 mg, 28.3 µmol) in NH_4_OAc (0.2 M, pH 4.4, 0.84 mL,) was added a solution of ^nat^Ga(NO_3_)_3_·8 H_2_O (7.0 mg, 17.5 µmol) in H_2_O (0.14 mL) and the resulting mixture was heated to 80°C for 3 hr. After cooling, the [^nat^Ga-**4**] complex was purified by repeated injections on a Waters ZQ HPLC-MS with a Waters Atlantis^®^ T3 C-18 10 µm preparative column (19 × 250 mm) using the following conditions; isocratic 20 mL/min , 4% ACN, 96% H_2_O with 0.01% TFA modifier, collecting the peak at t = 7.8 min by the ES +ve chromatographic trace. The combined fractions were reduced by rotary evaporation, and freeze-dried yielding a clear solid (20 mg). ^1^H-NMR (CD_3_OD + 5% D_2_O): δ (ppm) 1.95 (m, 1H), 2.14 (m, 1H), 2.25 (m, 2H), 3.25–3.45 (complex m, 12H), 3.51 (t, *J* = 7 Hz, 2H), 3.64 (m, 6H), 3.58 (m, 2H), 3.73 (br s, 4H), 3.8–3.92 (m, 4H), 3.96 (s, 2H), 4.41 (m, 1H); ^13^C{^1^H}NMR (d_6_-DMSO): δ (ppm) 26.64, 28.96, 31.24, 38.43, 51.17, 53.89, 54.28, 56.47, 56.53, 59.62, 60.50, 61.44, 68.81, 69.33, 69.67, 70.16, 166.52, 169.21, 169.37, 169.93, 173.05, 173.49. ESI-MS m/z [M+H]^+^ 744.25, 746.22, [M+Na]^+^ 766.20, 768.14, [M-H]^−^ 742.27, 744.27. HR-MS (TOF MS ES^+^): 766.2151 calculated for [C_27_H_44_N_7_O_13_^69^GaNa]^+^, found 766.2047, HR-MS (TOF MS ES^−^) 744.2166 calculated for [C_27_H_43_N_7_O_13_^71^Ga]^−^, found 744.2166; HPLC-ELSD purity 97.9% Method A (6.3 min).

*Synthesis of (S)-tert-butyl 5-amino-2-(6-(tert-butoxycarbonylamino)hexanamido)-5-oxopentanoate* (**5**). 6-(Boc-amino)hexanoic acid (1.0 g, 4.32 mmol), l-glutamine *t*-butyl ester hydrochloride (1.03g, 4.32 mmol) HOBt (0.64 g, 4.75 mmol), EDCi (0.91 g, 4.75 mmol) and DIPEA (1.4 mL) were stirred in anhydrous DMF (50 mL) under N_2_ atmosphere at 0 °C for 2hr. The reaction was allowed to proceed overnight at RT. The solution was reduced *in vacuo* and EtOAc (50 mL) was added. The resulting organic phase was washed with saturated NaHCO_3_ solution (2 × 20 mL), H_2_O (2 × 10 mL) and brine (2 × 20 mL). The solution was dried over Na_2_SO_4_ and evaporated to a white foam (1.685 g) ^1^H-NMR (d_6_-DMSO): δ (ppm) 1.22 (m, 2H), 1.35–1.41 (2 × overlapped m, 18H), 1.43–1.53 (complex m, 2H), 1.66–1.76 (m, 1H), 1.83–1.92 (m, 1H), 2.06–2.13 (2 ×overlapped t, *J* = 7.7 Hz, 7.1 Hz, _,_4H), 2.87 (q, *J* = 6.8 Hz, 2H), 4.02–4.07 (m, 1H), 6.70–6.79 (br m, 2H), 7.20–7.34 (br s, 1H), 8.04 (d, *J* = 7.5 Hz, 1H); ^13^C{^1^H}NMR (d_6_-DMSO): δ (ppm) 24.98, 25.94, 26.69, 27.65, 28.28, 29.26, 31.23, 35.0, 40.1 (discerned from residual d_6_-DMSO in HSQC spectrum), 52.28, 77.31, 80.32, 155.57, 171.33, 172.28, 173.33; ESI-MS m/z [M+H]^+^, 416.20 [M+Na]^+^ 438.15; HR-MS (TOF MS ES^+^): 416.2761 calculated for [C_20_H_38_N_3_O_6_]^+^, found 416.2770; HPLC-ELSD purity 100% Method B (11.8 min).

*Synthesis of (S)-5-amino-2-(6-aminohexanamido)-5-oxopentanoic acid· TFA salt* (**6**) [36]. Compound **5** (0.35 g, 0.84 mmol) was stirred in neat TFA (3.4 mL) for 2 h at RT. The TFA was evaporated off *in vacuo*, and H_2_O was added (5 mL). The aqueous phase was washed with EtOAc (2 × 5 mL) and reduced *in vacuo* to a colorless oil (508 mg), ^1^H-NMR (D_2_O): δ (ppm) 1.35–1.44 (complex m, 2H), 1.61–1.74 (overlapped m, 4H), 1.95–2.05 (complex m, 1H), 2.12 - 2.22 (complex m, 1H), 2.30–2.40 (overlapped t, *J* = 7.7 Hz, 7.4 Hz, _,_4H), 2.99 (t, *J* = 7.5 Hz, 2H), 4.31 (dd, *J* = 5.1 Hz, 9.0 Hz, 1H); ^13^C{^1^H}NMR (D_2_O): δ (ppm) 25.19, 25.64, 26.98, 27.01, 31.86, 35.62, 39.90, 52.80, 112.63^*^, 115.53^*^, 118.43^*^, 121.34^*^, 163.06^*^, 163.40^*^, 163.76^*^, 164.11^*^, 175.81, 177.53, 178.57 (^*^CF_3_COOD); ESI-MS m/z [M+H]^+^, 260.11; HR-MS (TOF MS ES^+^): 260.1610 calculated for [C_11_H_22_N_3_O_4_]^+^, found 260.1600; HPLC-ELSD purity 96.2% Method A (6.1 min).

*Synthesis of (S)-2,2',2''-(10-(2-(6-(4-amino-1-carboxy-4-oxobutylamino)-6-oxohexylamino)-2-oxo-ethyl)-1,4,7,10-tetraazacyclododecane-1,4,7-triyl)triacetic acid* *·* *HCOOH* (**7**), *‘DOTAMA-C_6_-Gln’.* To a solution of DOTA-NHS ester (121.6 mg, 123.3 µmol) in DMSO (1.1 mL) and ACN (0.4 mL) was added compound **6** (70 mg, 187.7 µmol) in DMSO (1 mL) and DIPEA (0.15 mL) and stirred at RT overnight. The solvent was evaporated off *in vacuo* and the resulting residue was diluted in 30% ACN in H_2_O (1.5 mL) and was purified by repeated injections on a Waters ZQ HPLC-MS with a Waters Atlantis^®^ T3 C-18 10 µm preparative column (19 × 250 mm) using the following conditions; isocratic 20 mL/min, H_2_O with 0.2% HCOOH modifier, collecting the peak at t = 8.3 min by the ES +ve chromatographic and ELSD traces. The combined fractions were reduced by rotary evaporation, and freeze dried yielding a clear solid (102.5 mg,). ^1^H-NMR (D_2_O): δ (ppm) 1.25–1.37 (m, 2H), 1.52 (m, 2H), 1.62 (m, 2H), 1.92–2.03 (m, 1H) 2.11–2.22 (m, 1H), 2.31 (t, *J* = 7.4 Hz, 2H), 2.37 (t, *J* = 7.4 Hz, 2H), 3.0–3.25 (br m, 10H), 3.25–3.61 (br m, 12H), 3.75–3.9 (br m, 4H) 4.31 (dd, *J* = 5Hz, *J* = 9Hz 1H); ^13^C{^1^H}NMR (D_2_O): δ (ppm) 25.54, 26.23, 27.40, 28.58, 32.07, 36.03, 39.96, 47.38, 48.98, 51.26, 51.89, 53.50, 54.26, 56.17; ESI-MS m/z [M+H]^+^ 646.34, [M−H]^−^ 644.31; HR-MS (TOF MS ES^+^): 646.3412 calculated for [C_27_H_48_N_7_O_11_]^+^, found 646.3395; HPLC-ELSD purity 96% Method A (7.7 min); Chiral purity >98%.

*Synthesis of ^nat^Ga(III)DOTAMA-C_6_-Gln· TFA salt* (^nat^Ga-**8**). To a stirred, buffered solution of compound **7** (29.1 mg, 39.65 µmol) in NH_4_OAc (0.2 M, pH 4.4, 2.0 mL,) and ACN (0.5 mL) was added a solution of ^nat^Ga(NO_3_)_3_. 8 H_2_O (10.0 mg, 25 µmol) in H_2_O (0.2 mL) and the resulting mixture was heated to 75 °C for 3 h. After cooling, the [^nat^Ga-***8***] complex was purified by repeated injections on a Waters ZQ HPLC-MS with a Waters Atlantis^®^ T3 10 µm C-18 preparative column (19 × 250 mm) using the following conditions; isocratic 20 mL/min, 7% ACN, 93% H_2_O (0.1% TFA), collecting the peak at t = 7.5 min by the ES +ve chromatographic trace. The combined fractions were reduced by rotary evaporation, and freeze-dried yielding a clear solid (25.25 mg,).^1^H-NMR (d_6_-DMSO): δ (ppm) 1.26 (m, 2H), 140 (m, 2H), 1.49 (m, 2H), 1.74 (m, 1H), 1.93 (m, 1H), 2.11 (m, 4H), 3.05 (m, 2H), 3.10–3.36 (complex m, 12H), 3.47–3.81 (complex m, 12H), 4.14 (m, 1H), 6.74 (br s, 1H), 7.26 (br s, 1H), 8.02 (d, *J* = 7.7 Hz, 1H), 8.32 (t, *J* = 5.5 Hz, 1H); ^13^C{^1^H}NMR (d_6_-DMSO): δ (ppm) 24.81, 25.98, 26.81, 28.61, 28.96, 31.35, 34.90, 38.35, 51.44, 53.92, 54.37, 56.50, 56.55, 59.62, 60.50, 61.51, 114.80, 117.74, 157.74, 158.08, 166.16, 169.19, 169.91, 172.18, 173.41, 173.52; ESI-MS m/z [M+H]^+^ 712.24, 714.19, [M+Na]^+^ 734.22, 736.13, [M−H]^−^ 710.29, 712.30; HR-MS (TOF MS ES^+^): 734.2252 calculated for [C_27_H_44_N_7_O_11_^69^GaNa]^+^, found 734.2280 (TOF MS ES^−^)710.2276 calculated for [C_27_H_43_N_7_O_11_^69^Ga]^−^, found 710.2279; HPLC-ELSD purity 99.39% Method A (8.7 min).

*Optical activity assessment of ligands* **3** *and* **7***. DOTAMA-PEG_2_-Gln* **3** or *DOTAMA-C_6_-Gln* **7** (1 mg) was dissolved in HCl (4 M, 0.3 mL) and heated for 3 h at 60 °C. The resulting solution was dried *in vacuo*. The solution was re-suspended in water and an aliquot (10 µL) was injected into an HPLC with a Phenomenex Chirex^®^ 3126 d-penicillamine LC column (150 × 4.6 mm) column heated to 40 °C, with 10% MeOH, 90% 2 mM CuSO_4_ running at 1.5 mL/min, with monitoring at 254 nm. Retention times were compared with injections of d- + l-glutamic acid under the same chromatographic conditions. Both ligand hydrolysis products gave rise to peaks at approximately 19.5 min, which corresponded to the l-enantiomer. The d-glutamic acid enantiomer had a higher retention of approximately 21.5 min, of which none could be reliably discerned from the wavy baseline recorded.

### 3.3. Radio-gallium Chemistry

#### 3.3.1. Radio-Synthesis of [^67^Ga-**4**] and [^67^Ga-**8**] and Formulation

General Radiolabelling Conditions: To a 2.0 mL microcentrifuge tube was added aqueous purified ^67^GaCl_3_ in 0.1M HCl, (0.66–1.0 mL, 209–274 MBq), 1M HEPES (330–500 μL). The pH of the solution was checked by spotting a pH indicator test strip with ~5 μL of solution; additional 1.0 M HCl (1–10 μL) or 1.0 M KOH (1–10 μL) was added to adjust the pH to the desired value, pH ~4. To this solution was added 15–25 µL of concentrated ligand (**3** or **7**) solution (1.0 mM). The vial was then capped and the mixture heated at 95 °C for 10 minutes after which it was allowed to cool to room temperature. A portion of the solution was analysed by HPLC and the reaction parameters are shown in Table 1.

**Table 1:** ^67^Ga Radiolabelling Parameters.

Complex Formed ^(& reacti^^o^^n ID)^	Ligand	Ligand quantity (nmol)	^68^Ga activity (MBq)	Radiochemical Purity (%)
[^67^Ga-4] ^3^°	3	1	2.7	82.4
[^67^Ga-8] ^26^	7	1	1.5	91.9
[^67^Ga-8] ^28^	3	2	1.6	98.5
[^67^Ga-8] ^27^	7	2	1.6	100
[^67^Ga-8] ^29^	7	2	3.2	100
[^67^Ga-4] ^31^	3	5	5.1	100
[^67^Ga-4] ^42^	3	5	36.6	100
[^67^Ga-4] ^44^	3	5	61.3	97.1
[^67^Ga-4] ^45^	3	5	101	96.2
[^67^Ga-8] ^25^	7	5	1.9	100
[^67^Ga-8] ^43^	7	5	61	97.2
[^67^Ga-8] ^46^	7	5	114	90.7
[^67^Ga-8] ^23^	7	10	1.8	100
[^67^Ga-8] ^47^	7	10	25.6	93.2
[^67^Ga-4] ^48^	3	15	260	96.1
[^67^Ga-4] ^49^	3	15	252	100
[^67^Ga-4] ^52^	3	15	232	100
[^67^Ga-4] ^54^	3	15	229	100
[^67^Ga-8] ^53^	7	15	266	100
[^67^Ga-8] ^55^	7	15	209	100
[^67^Ga-4] ^5^°	3	25	274	94.1
[^67^Ga-8] ^51^	7	25	248	100

HPLC Separation: The radiolabelled compound was purified (separated from reagents and remaining ligand) using a semi-preparative HPLC system. Two separate runs were performed for each reaction, typical injection volumes onto the system were, 0.7 to 1.0 mL. Separation of the achieved employing a Waters Atlantis^®^ T3 C-18 5 μm semi-preparative column (10 × 250 mm) under isocratic conditions (3 mL/min; 94/6, 10 mM aqueous NH4OAc buffered to pH ~5 with AcOH/MeOH), unreacted ‘^67^Ga’ eluted at the solvent front while the ^67^Ga-complex and ligand eluted at 9.6 min and 11.3 min for [^67^Ga-**4**] and **3** and 12.5 min and 15.5 min for [^67^Ga-**8**] and **7**. Fractions of the [^67^Ga-**4**] were typically collected between 9–10 min and for [^67^Ga-**8**] between 12–13.5 min. Fractions were collected into 20 mL plastic vials. Recoveries were typically in excess of 90%.

Concentration and Reformulation: The column eluates were diluted to ~20 mL with deionised H_2_O and manually adsorbed onto Waters Oasis^®^ MAX plus cartridges. The columns were washed with deionised H_2_O (~20 mL) and the product eluted with 10× concentrated DPBS buffer (plus Mg^2+^ and Ca^2+^, 500 μL). Overall activity recoveries generally exceed 80%. The product eluates were diluted up to 5 mL with deionised H_2_O and used for biological studies. Typically, 100–200 µL of this solution was retained for QC analysis and buffer stability studies. 

#### 3.3.2. Stability of [^67^Ga-**4**] and [^67^Ga-**8**] Radio-Complexes

The solutions of radio-complexes, [^67^Ga-**4**] and [^67^Ga-**8**] were assayed for stability in PBS buffer over the period of 7 days by repeated injections (25 or 50 µL) of the products onto radio-HPLC systems monitoring the product peak and free ^67^Ga. Chromatography conditions are as described above.

#### 3.3.3. Radio-Synthesis of [^68^Ga-**4**] and [^68^Ga-**8**]

General Conditions: To a 1.5 mL microcentrifuge tube was added aqueous purified [^68^Ga(OH_4_)]^−^ in 0.5M KOH, (500 μL, 27.8–28 MBq), 1M HEPES (250 μL) and HCl (4M, 80 µL). The pH of the solution was checked by spotting a pH indicator test strip with ~5 μL of solution; additional 1.0 M HCl (1–10 μL) or 1.0 M KOH (1–10 μL) was added to adjust the pH to the desired value, pH ~4. To this solution was added 10 µL of concentrated ligand (**7** or **8**) solution (1 mM). The vial was then capped and the mixture heated at 95 °C for 10 min. The solution was allowed to cool to room temperature before a 50 µL sample was withdrawn and injected onto a radio-HPLC system for analysis. Results of the labelling are presented in Table 2.

**Table 2:** ^68^Ga Radiolabelling Parameters.

Complex	Ligand	Ligand quantity (nmol)	^68^Ga activity (MBq)	Radiochemical Purity (%)
[^68^Ga-4]	3	5	0.97	91.8
[^68^Ga-4]	7	5	0.79	93.6
[^68^Ga-4]	3	30	0.93	96.6
[^68^Ga-8]	7	30	0.80	97.9
[^68^Ga-4]	3	10	28.0	98.4
[^68^Ga-8]	7	10	27.8	93.3

### 3.4. Cell Cultures

Cell lines MDA, MCF-7, A375 and PC3 were maintained sub confluent in 175 cm^2^ tissue culture flasks containing 25 mL of Roswell Park Memorial Institute (RPMI) media supplemented with 10% foetal bovine serum, l-glutamine (2 mM), streptomycin (100 µg/mL) and penicillin (100 units).

### 3.5. Cell Uptake Studies

All uptake experiments were completed in triplicate for each compound on each cell line. Cells were trypsinised, seeded into 24 well plates at 2.5 × 10^5^ cells/mL in 500 µL of media and left to attach overnight in a CO_2_ incubator. Each well was washed twice with DPBS (1 mL) and then with DPBS with Ca and Mg added (500 µL) and the cells placed back into the incubator for 45 min. The ^67^Ga labelled compound was formulated to a concentration of 0.74 MBq/mL in DPBS with Ca and Mg. To start the incubation the wells were thoroughly aspirated and 500 µL of the formulated ^67^Ga compound added and placed into a 36 °C incubator. Incubations were terminated at the appropriate time point by first aspirating the well completely, washing twice with ice cold DPBS (1 mL) and lysing the cells with aqueous NaOH (0.2M, 500 µL). All time points were done in triplicate. The lysate was collected into glass Kimble tubes and counted on a gamma counter. An aliquot of the formulated ^67^Ga compound was counted alongside the lysates and the percentage the total activity internalised by the cells, as measured in the lysates, was calculated for each time point. To account for loss of activity due to non-specific interactions, a 500 µL aliquot of formulated ^67^Ga compound was incubated, in triplicate, in a clean well of a 24 well plate which was then treated as per the longest time point for each of the cell incubations.

### 3.6. Competitive Inhibition Studies in Cells

Uptake inhibitors, l-glutamine, BCH and MeAIB were prepared on the day of the experiment to a concentration of 12.5 mM in DPBS with Ca and Mg. Cell lines were prepared as above. ^67^Ga compounds were formulated to 3.7 MBq/mL in DPBS with Ca and Mg. Incubations were conducted for each cell line with four treatment groups done in triplicate; Control, l-glutamine, BCH and MeAIB. Incubations were started by completely aspirating the well, adding 400 µL of the relevant treatment and 100 µL of formulated [^67^Ga-**4**] or [^67^Ga-**8**] radio-complex. The incubation was allowed to proceed for 60 min at 36 °C after which it was terminated and analysed as above.

## 4. Conclusions

A number of novel precursor molecules and two novel gallium complexes have been synthesised by conventional peptide coupling methodologies and purified by HPLC-MS means. Initial attempts to produce the ^67^Ga complexes from the commercially available ^67^Ga citrate were unreliable, so conversion of the citrate salt to the ^67^GaCl_3_ was performed which gave reliable and high yields in the HEPES buffered reactions at pH ~4 (>90%) using nanomole amounts of ligand. The ^67^Ga- radiolabeled complexes were found to be stable for at least 1 week in PBS buffer. The PET relevant ^68^Ga radio-complex analogues were also produced in high yield (>91%) by the same method but starting from the [^68^Ga(OH)_4_]^−^ species eluted by the generator The ^67^Ga complexes were tested *in vitro* against four tumor cell lines, with low uptake (<1%) observed. The ^67^Ga complexes were also tested for their uptake *versus* l-glutamine, and two known amino acid transporter inhibitors, with results pointing to one complex, [^67^Ga-**4**], exhibiting uptake via a non targeted amino acid pathway. These results indicate that the Ga complexes do not mimic l-glutamine and therefore, the potential for ^68^Ga- glutamine based PET tracers must be re-evaluated before they could compete with simpler non-metallic PET analogues.
